# P-2358. Viral etiologies of bronchiolitis, pneumonia, and croup in children less than 5 years old, New Vaccine Surveillance Network, 2016-2022

**DOI:** 10.1093/ofid/ofae631.2509

**Published:** 2025-01-29

**Authors:** Zheyi Teoh, Ariana Perez, Christina M Quigley, Amy Ostrow, Chelsea Rohlfs, Christopher J Harrison, Eileen J Klein, Geoffrey A Weinberg, Janet A Englund, John V Williams, Julie A Boom, Leila C Sahni, Marian G Michaels, Natasha B Halasa, Peter G Szilagyi, Rangaraj Selvarangan, Heidi L Moline, Mary A Staat

**Affiliations:** Seattle Children's Hospital, Seattle, Washington; CDC, Avondale Estates, Georgia; Cincinnati Children's Hospital Medical Center, Cincinnati, Ohio; Cincinnati Children's Hospital Medical Center, Cincinnati, Ohio; Cincinnati Children's Hospital, Cincinnati, Ohio; Children's Mercy Hospital, Kansas City, Missouri; University of Washington School of Medicine, Seattle, Washington; University of Rochester School of Medicine & Dentistry, Rochester, NY; Seattle Children’s Hospital, Seattle, Washington; University of Pittsburgh, Pittsburgh, Pennsylvania; Texas Children’s Hospital, Houston, Texas; Baylor College of Medicine and Texas Children’s Hospital, Houston, Texas; UPMC Children's Hospital of Pittsburgh, Pittsburgh, Pennsylvania; Vanderbilt University Medical Center, Nashville, TN; UCLA School of Medicine, Agoura Hills, California; Children’s Mercy Kansas City, Kansas City, Missouri; Centers for Disease Control and Prevention, Atlanta, Georgia; Cincinnati Children’s Hospital Medical Center, Cincinnati, Ohio

## Abstract

**Background:**

Acute respiratory illness (ARI) is a leading cause of medically attended visits among children. A multi-season, multicenter, prospective evaluation of viral etiologies for different ARI syndromes is lacking. We describe the epidemiology of respiratory viruses (RV) detected among children with bronchiolitis, pneumonia, and croup in the New Vaccine Surveillance Network (NVSN).

**Table 1.**
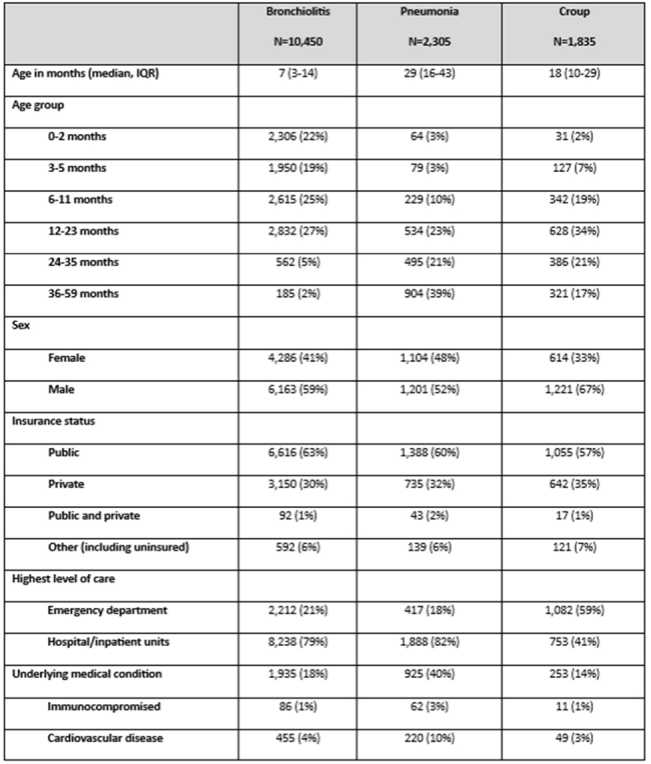
Demographic characteristics of children less than 5 years with different acute respiratory illness syndrome (n=14,590), New Vaccine Surveillance Network, 2016-2022

**Methods:**

NVSN is a population-based, prospective, active surveillance network conducted at 7 pediatric hospitals. During 2016-2022, children < 5 years old with ARI were enrolled in the emergency department (ED) or inpatient unit. Respiratory samples obtained at enrollment were tested by RT-PCR for 8 RV (Figure 1). Demographic and clinical data were collected by caregiver surveys and chart review. ICD-10 codes were used to identify cases of bronchiolitis, pneumonia, and croup; cases with multiple ARI syndromes of interest were excluded. Descriptive statistics were used to summarize the proportion of viral detections by ARI syndrome and age group.

**Figure 1.**
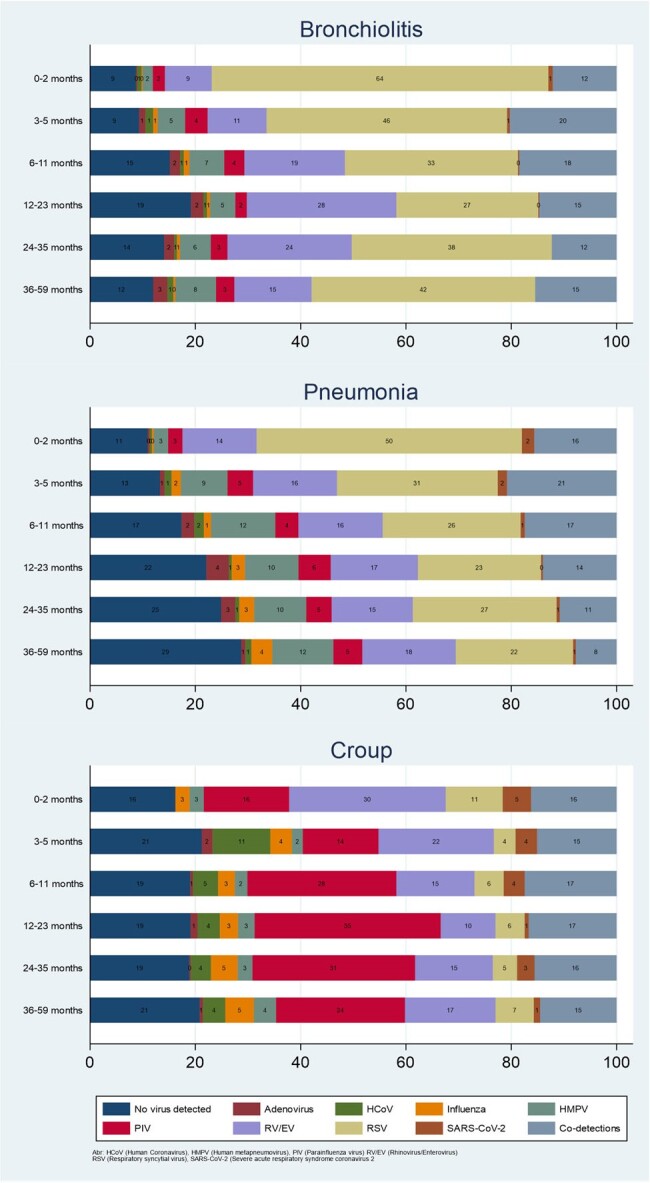
Respiratory virus detection across different acute respiratory illness syndromes, stratified by age , New Vaccine Surveillance Network, 2016-2022

**Results:**

Of 44,194 children enrolled, 14,590 were diagnosed with one ARI syndrome of interest, including 10,450 (71.6%) with bronchiolitis, 2,305 (15.8%) with pneumonia, and 1,835 (12.6%) with croup (Table 1). Of 14,590 children with one ARI syndrome, 12,138 (83%) had at least one virus detected. Among children with bronchiolitis or pneumonia, respiratory syncytial virus (RSV) and rhinovirus/enterovirus (RV/EV) were the most commonly detected viruses (Figure 1), whereas among children with croup, parainfluenza viruses (PIV) and RV/EV were most commonly detected. Among hospitalized children, children with pneumonia had the highest proportion of supplemental oxygen administration (69%), mechanical ventilation (7%), and death (0.3%). No deaths were reported for children with bronchiolitis or croup.

**Table 2.**
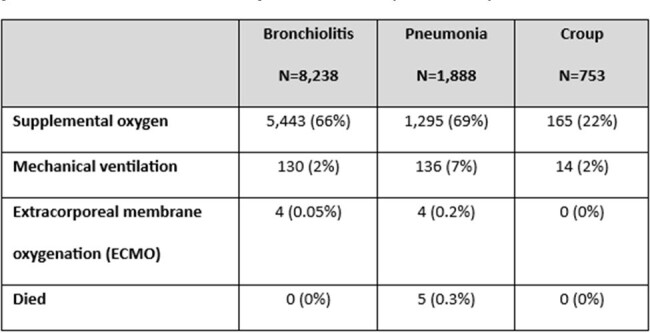
Severity and respiratory support across different acute respiratory illness syndromes evaluated in inpatient units (n=10,879), NVSN, 2016-2022

**Conclusion:**

From 2016-2022, RSV was commonly detected among children with bronchiolitis or pneumonia in the ED and inpatient settings and PIV was commonly detected among children with croup; RV/EV was commonly detected for all syndromes. Respiratory virus detections among common ARI diagnoses may evolve with the introduction of new respiratory virus prevention products underscoring the importance of continued evaluation.

**Disclosures:**

Christopher J. Harrison, MD, GSK: Grant/Research Support|Medscape: Honoraria|Merck: Grant/Research Support|Pfizer: Grant/Research Support|UpToDate: Honoraria Geoffrey A. Weinberg, MD, Inhalon: Advisor/Consultant|Merck & Company: Honoraria for textbook chapter preparation Janet A. Englund, MD, Abbvie: Advisor/Consultant|AstraZeneca: Advisor/Consultant|AstraZeneca: Grant/Research Support|GlaxoSmithKline: Advisor/Consultant|GlaxoSmithKline: Grant/Research Support|Meissa Vaccines: Advisor/Consultant|Merck: Advisor/Consultant|Pfizer: Board Member|Pfizer: Grant/Research Support|Pfizer: Speaker at meeting|SanofiPasteur: Advisor/Consultant|Shinogi: Advisor/Consultant Natasha B. Halasa, MD, MPH, Merck: Grant/Research Support Rangaraj Selvarangan, BVSc, PhD, D(ABMM), FIDSA, FAAM, Abbott: Grant/Research Support|Abbott: Honoraria|BioMerieux: Grant/Research Support|Cepheid: Grant/Research Support|Diasorin: Grant/Research Support|GSK: Advisor/Consultant|Hologic: Grant/Research Support|Luminex: Grant/Research Support|Qiagen: Grant/Research Support Mary A. Staat, MD, MPH, Cepheid: Grant/Research Support|Merck: Grant/Research Support|Pfizer: Grant/Research Support|Up-To-Date: Honoraria

